# Diet and Pediatric Functional Gastrointestinal Disorders in Mediterranean Countries

**DOI:** 10.3390/nu14112335

**Published:** 2022-06-02

**Authors:** Caterina Strisciuglio, Sabrina Cenni, Maria Rosaria Serra, Pasquale Dolce, Sanja Kolacek, Sara Sila, Ivana Trivic, Michal Rozenfeld Bar Lev, Raanan Shamir, Aco Kostovski, Alexandra Papadopoulou, Eleftheria Roma, Christina Katsagoni, Danijela Jojkic-Pavkov, Angelo Campanozzi, Elena Scarpato, Erasmo Miele, Annamaria Staiano

**Affiliations:** 1 Department of Woman, Child and General and Specialized Surgery, University of Campania “Luigi Vanvitelli”, 81100 Naples, Italy; caterina.strisciuglio@unicampania.it (C.S.); sabrina.cenni@unicampania.it (S.C.); 2 Department of Translational Medical Science, Section of Pediatrics, University of Naples “Federico II”, 80138 Naples, Italy; mrserra.studio@gmail.com (M.R.S.); elenascarpato@hotmail.it (E.S.); erasmo.miele@unina.it (E.M.); 3 Department of Public Health, University of Naples “Federico II”, 80138 Naples, Italy; pasquale.dolce@unina.it; 4 Referral Center for Pediatric Gastroenterology and Nutrition, Children’s Hospital University of Zagreb Medical School, 10000 Zagreb, Croatia; sanja.kolacek@gmail.com (S.K.); sara.sila0810@gmail.com (S.S.); ivana.trivic.0@gmail.com (I.T.); 5 Institute of Gastroenterology, Nutrition and Liver Diseases, Schneider Children’s Medical Center, Sackler Faculty of Medicine, Tel-Aviv University, Tel Aviv 69978, Israel; michal.barlev@gmail.com (M.R.B.L.); raanan@shamirmd.com (R.S.); 6 Faculty of Medicine, University Children Hospital, 1000 Skopje, North Macedonia; acokos@gmail.com; 7 Division of Gastroenterology and Hepatology, First Department of Pediatrics, University of Athens, “Agia Sofia” Children’s Hospital, 11527 Athens, Greece; office.alexandra.papadopoulou@gmail.com; 8 First Department of Pediatrics, Medical School, National and Kapodistrian University of Athens, Mikras Asias 75, 11527 Athens, Greece; roma2el@otenet.gr; 9 Department of Clinical Nutrition, “Agia Sofia” Children’s Hospital, 11527 Athens, Greece; christina.katsagoni@gmail.com; 10 Department of Paediatrics, Institute for Child and Youth Health Care of Vojvodina, Medical Faculty Novi Sad, 21000 Novi Sad, Serbia; jojkicd@gmail.com; 11 Pediatrics, Department of Medical and Surgical Sciences, University of Foggia, 71100 Foggia, Italy; angelo.campanozzi@unifg.it

**Keywords:** functional gastrointestinal disorders, FGIDs, FODMAP, Mediterranean diet

## Abstract

Background: The increased intake of FODMAP (fermentable oligosaccharides, disaccharides, monosaccharides, and polyol) rich foods has been suggested as a possible trigger of functional gastrointestinal disorders (FGIDs). Despite the high FODMAP content, the Mediterranean diet (MD) appears to have beneficial effects on health. Our aim was to evaluate whether the prevalence of FGIDs in different Mediterranean countries may be influenced by FODMAP consumption and adherence to the MD. Methods: A school-based, cross-sectional, multicenter study was performed in six countries in the Mediterranean area: Croatia, Greece, Israel, Italy, Macedonia, and Serbia. Subjects 4-18 years were examined in relation to their eating habits and the presence of FGIDs, using Rome IV criteria, 3-day food diaries and Mediterranean Diet Quality Index in Children and Adolescents (KIDMED) questionnaires. Results: We enrolled 1972 subjects between 4 and 9 years old (Group A), and 2450 subjects between 10 and 18 years old (Group B). The overall prevalence of FGIDs was 16% in Group A and 26% in Group B. FODMAP intake was significantly different among countries for both age groups. In both groups, no significant association was found between FGIDs and FODMAPs. Adherence to the MD in all countries was intermediate, except for Serbia, where it was low. In both groups, we found a statistically significant association between FGIDs and the KIDMED score (Group A: OR = 0.83, *p* < 0.001; Group B: OR = 0.93, *p* = 0.005). Moreover, a significant association was found between the KIDMED score and functional constipation (Group A: OR = 0.89, *p* = 0.008; Group B: OR = 0.93, *p* = 0.010) and postprandial distress syndrome (Group A: OR = 0.86, *p* = 0.027; Group B: OR = 0.88, *p* = 0.004). Conclusions: Our data suggest that the prevalence of FGIDs in the Mediterranean area is not related to FODMAP consumption, whereas adherence to the MD seems to have a protective effect.

## 1. Introduction

Functional gastrointestinal disorders (FGIDs) are defined as a variable combination of chronic or recurrent gastrointestinal symptoms that are age dependent and not explained by structural or biochemical abnormalities. The pathogenesis of FGIDs remains unclear, although various mechanisms have been proposed, such as gut dysmotility, visceral hypersensitivity, gut immune abnormalities, small intestinal bacterial overgrowth with impaired barrier function, psychosocial factors and a dysregulated gut–brain axis [1,2].

In addition, diet seems to be an important factor in the etiopathogenesis of irritable bowel syndrome (IBS) [3]. Nutrients can affect gastrointestinal (GI) motility, sensitivity, barrier function, and the gut microbiome, modulating atypical mechanisms in the gut [3]. Moreover, it has been reported that at least two-thirds of adult patients with IBS, as well as two-thirds of children with FGIDs, perceive their gastrointestinal symptoms to be food related, making dietary management an important aspect in IBS management [3]. Recently, more interest has been focused on low fermentable oligosaccharides, disaccharides, monosaccharides, and polyol (FODMAP) diets, in which the intake of these fermentable carbohydrates is reduced [4]. The increasing availability in Western countries of foods rich in FODMAPs has brought up the question of their role as a possible trigger of FGIDs. There are various ways in which FODMAPs may lead to GI symptoms, including bowel distension and alterations in the gut microbiome, GI endocrine cells, immune function, and/or the intestinal barrier [5]. Recently, Chumpitazi et al. demonstrated that in children with IBS, a low-FODMAP diet decreases abdominal pain frequency [6]; other studies showed the promising effects of low-FODMAP diets in reducing functional GI symptoms [7]. In contrast, emerging evidence supports the hypothesis that a Mediterranean diet (MD), which is rich in FODMAPs, may be beneficial for FGIDs [8,9]. The MD is considered as a complex set of eating habits adopted in countries bordering the Mediterranean Sea. The large consumption of fruit and vegetables rich in antioxidants, increased intake of omega 3 in fish and reduced consumption of saturated fats are the main factors that determine the beneficial health effects of the MD.

We have recently shown that in the Mediterranean area, the overall prevalence of FGIDs, according to Rome III criteria, was 20.7% in children between 4 and 10 years old and 26.6% in adolescents between 11 and 18 years old [10]. We found significant differences in the prevalence of FGIDs among European countries; however, the reasons for these differences are still unknown.

These results suggest that different environments, gut microbiomes, dietary habits and genetic backgrounds among the involved countries may contribute to the multifactorial etiopathogenesis of FGIDs. For these reasons, it is not possible to generalize the results obtained from single-country studies. Therefore, we sought to determine whether the prevalence of FGIDs in Mediterranean countries varies according to FODMAP intake, adherence to the MD or other specific dietary components.

## 2. Methods

A multicenter, cross-sectional study was performed in 6 countries in the Mediterranean area—Croatia, Greece, Israel, Italy, Macedonia, and Serbia—from October 2019 to September 2020. In the 6 countries of the Mediterranean area, public nursery, primary and secondary schools were randomly selected to obtain a nationwide sample. A meeting was organized to explain this study to parents/legal guardians and their children. Informed consent was obtained at that time. Questionnaires were completed in the presence of the study staff, who was available for clarifications. We enrolled all healthy subjects between 4 and 18 years old. Children were included if consent was provided and in the absence of alarming symptoms suggestive of a gastrointestinal organic disease. Children and adolescents with known clinical pathologies in their medical history were excluded.

This study was approved by the Ethics Committees of the coordinating center (University of Campania “Luigi Vanvitelli” of Naples) and of the other participating centers, and was conducted in accordance with the Declaration of Helsinki and Guidelines for Good Clinical Practice (registration number 2018/685). All data were collected anonymously and were entered into a Microsoft Excel database specifically designed for a previous study [11].

### 2.1. Rome Questionnaires

We used the Rome IV Diagnostic Questionnaire on Pediatric Functional Gastrointestinal Disorders (R4PDQ), specifically designed to diagnose FGIDs in children and adolescents [12]. R4PD has been translated for non-English-speaking countries, validated and approved by the Rome Foundation as described in our previous work [11].

The questionnaire was presented in 2 forms: Child: Self-report form for Children and Adolescents (10 years and older), and R4PDQ—Child: Parent-report form for Children (4 years and older).

Using the R4PDQ scoring system, an algorithm was created to diagnose FGIDs.

### 2.2. Dietary Intake

Dietary intake was assessed using a 3-day food diary record [13]. Parents and adolescents were asked to record every meal they ate, specifying the amounts consumed (in household measures), cooking methods and brands of food products.

To analyze any changes in the diet, food diary records were reported for 2 days of the week and one day of the weekend. Data from the diaries were analyzed for energy, macronutrients and micronutrients and FODMAP intake, using nutrient analysis software Winfood^®^ PRO 3.9.x (MedimaticaSurl, Teramo, Italy) and PRODI software.

Daily FODMAP intake quantification was performed for total fructan, galacto-oligosaccharides (stachyose and raffinose), polyols (sorbitol and mannitol), fructose, and lactose. With the exception of fructans and galactans, the rest of the FODMAP concentrations (g per 100 g of food) were obtained from the Winfood and Prodi software. The fructan and galactans values of the various foods have been extrapolated from the Food Composition Tables from the *Research Centre* for Agriculture and Environment *(RCAE)* relating to the foods most consumed in Mediterranean areas, and from the Complete Food Tables to standardize the data with other countries [14,15].

Adherence to the MD was evaluated using a specific questionnaire, the Mediterranean Diet Quality Index in Children and Adolescents (KIDMED), comprising 16 questions with scores ranging from 0 to 12 (>8 optimal; 4–7 intermediate; <3 very low adherence) [16]. The KIDMED questionnaire was answered by parents or adolescents.

### 2.3. Statistical Analysis

Quantitative variables were synthetized using the means ± standard deviations (SD) or the median (I quartile, III quartile), as deemed appropriate, while qualitative variables were described using absolute frequencies (percentages).

The prevalence of each disorder was presented with the corresponding 95% CI, which was obtained using the procedure first proposed by Clopper and Pearson [17].

Differences in the prevalence of disorders between men and women and among countries were assessed using the chi-square test or the Fisher exact test, as deemed appropriate.

The Kruskal–Wallis test was used to assess differences among countries in terms of FODMAP daily intake, since variable distributions were skewed. Dunn’s test was used for multiple comparisons, adjusting *p*-values with the Bonferroni method.

The association between FODMAPs, all macronutrient and micronutrient intakes, and the KIDMED score and FGIDs was assessed using, separately for each variable, multilevel logistic regression analysis considering countries as second-level units. Models with random slopes were compared to models with only random intercepts using likelihood-ratio tests.

Statistical significance was considered for *p*-values or corrected *p*-values less than 0.01. All statistical analyses were performed using R, a software environment for statistical computing. The glmer function implemented in the lme4 R package [18] was used for multilevel logistic regression analysis.

## 3. Results

A total of 4422 subjects, between 4 and 18 years, from 6 countries in the Mediterranean area participated in this survey.

Specifically, we included 1972 subjects between 4 and 9 years old (Group A: mean age 7.3 ± 1.6 y (range 4–9); girls [females (F)], 53%), and 2450 subjects between 10 and 18 years old (Group B: mean age 13.1 ± 2.3 y (range 10–17); F, 57.6%). The prevalence of all FGIDs defined according to Rome IV criteria in both study groups is summarized in Supplementary Table S1.

In subjects aged between 4 and 9 years old, the overall prevalence of FGIDs was 16% (F, 59.1%), with a statistically significant difference among countries (*p* < 0.001). The overall prevalence of FGIDs was 12% in Croatia, 18% in Greece, 33% in Israel, 26% in Italy, 12% in Macedonia and 8% in Serbia. In this age group, the overall prevalence of FGIDs did not significantly differ between genders. The most frequent disorders were functional constipation (FC) (7.46%), postprandial distress syndrome (PDS; 3.35%), and abdominal migraine (AM; 1.12%), IBS; 0.61%). The prevalence of all FGIDs in children between 4 and 9 years old and related valid cases are shown in Supplementary Table S2.

In subjects between 10 and 18 years old, the overall prevalence of FGIDs was 26% (F, 62.3%), with a statistically significant difference among countries (*p* < 0.001). The overall prevalence of FGIDs was 18% in Croatia, 16% in Greece, 50% in Israel, 26% in Italy, 33% in Macedonia and 18% in Serbia. In this age group, the overall prevalence of FGIDs did not significantly differ between sexes. The most frequent disorders were FC (13.7%), PDS (5.37%), AM (2.77%), epigastric pain syndrome (EPS; 1.17%) and IBS (1.11%). The prevalence of FGIDs in subjects between 10 and 18 years old and related valid cases in the different countries is shown in Supplementary Table S2.

### 3.1. FODMAP Intake

Regarding the FODMAP daily intake, we found statistically significant differences among the various countries included in this study in both groups (Table 1). In both groups, no significant association was found between FGIDs and FODMAPs. Additionally, there was no significant association found when considering FGIDs with the highest prevalence.

### 3.2. Macronutrients and Micronutrient Intake

The analysis of macronutrient and micronutrient intake showed very different dietary habits in the six countries included in this study (Table 2).

In Group A, no associations were found between FGIDs and both macronutrient and micronutrient intake. No significant associations were found between macronutrients and FGIDs, with the highest prevalence in Group A, whereas significant associations were found between PDS and vitamin E (OR 0.56, 95%CI [0.36; 0.82], *p* = 0.005) among micronutrients. In Group B, there were no associations found between FGIDs and both macronutrient and micronutrient intake, even when considering FGIDs with the highest prevalence. No associations were found between FGIDs, including FC, and dietary fiber among both groups. The effect of all macronutrients and micronutrients on FGIDs did not significantly vary from one country to another.

### 3.3. The Mediterranean Diet

**Figure 1:**
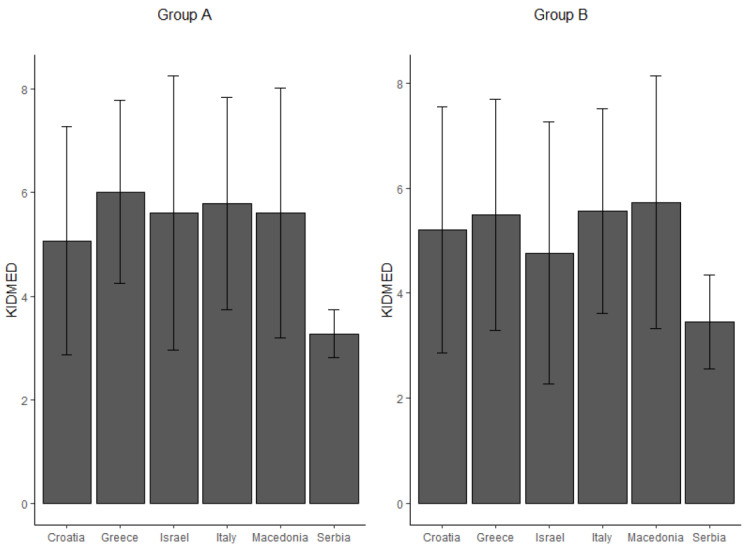
Adherence to the Mediterranean diet evaluated by the KIDMED in children (Group A) and adolescents (Group B).

**Figure 2:**
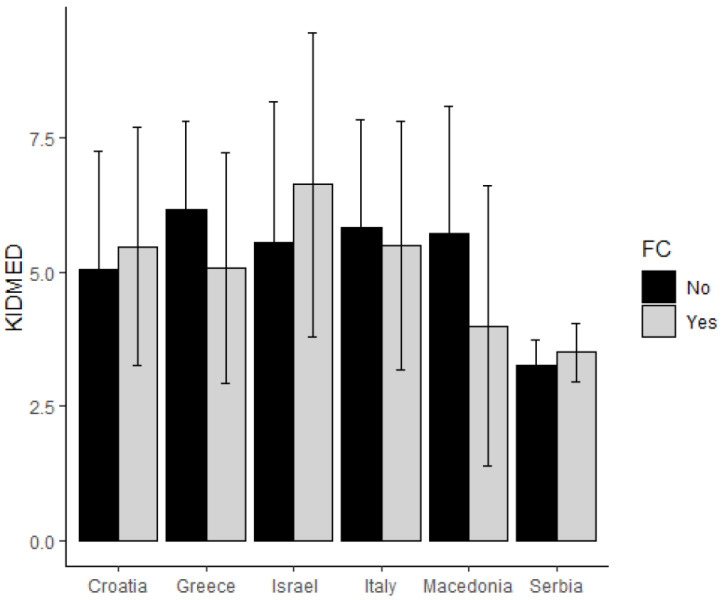
The distribution of adherence to the Mediterranean diet in Group A across countries for patients with functional constipation and without functional constipation.

Adherence to the MD was intermediate in all countries included, except for Serbia, where it was low and differences among countries were statistically significant (*p* < 0.001) in both groups (see Figure 1). In Group A, there was a statistically significant association between FGIDs and the KIDMED score (OR = 0.83, 95%CI [0.77; 0.90], *p* < 0.001), which did not significantly vary from one country to another (*p* = 0.048). Regarding FGIDs with the higher prevalence in this group, a significant association was found between the KIDMED score and FC (OR = 0.89, 95%CI [0.81; 0.97], *p* < 0.008), which significantly varied from one country to another (*p* = 0.008). Figure 2 shows the distribution of the adherence of the MD in Group A across countries for patients with FC and without FC. In Group B there was a statistically significant association between FGIDs and the KIDMED score (OR = 0.93, *p* = 0.005), which did not significantly vary from one country to another (*p* = 0.046). Regarding FGIDs with the higher prevalence in this group, we found a significant association between the KIDMED score and FC (OR = 0.93, 95%CI [0.88; 0.98], *p* = 0.010), which did not vary from one country to another (*p* = 0.173). Moreover, there was a significant association between the KIDMED score and PDS (OR = 0.88, 95%CI [0.81; 0.96], *p* = 0.004) which also did not significantly vary from one country to another (*p* = 0.999). There was no association between KIDMED scores and FODMAPs among both age groups.

## 4. Discussion

This school-based cross-sectional study recruited a large number of children and adolescents, nationwide, for each of the six included countries from the Mediterranean area. The aim was to evaluate if the prevalence of FGIDs is associated with the FODMAP content in the diet, with adherence to the MD, or with specific dietary components. The main findings of our study were that the prevalence of FGIDs was not related to FODMAPs, while the prevalence of FGIDs varied according to adherence to the MD, which showed a protective effect on the development of FGIDs.

In our study population, 16% of children between 4 and 9 years old and 26% of adolescents between 10 and 18 years old fulfilled the Rome IV criteria for at least 1 FGID. As in our previous study [10], we confirmed significant variations in the prevalence of some FGIDs among different European countries, although the reasons for these differences remain unclear. In respect to the diet of each country, there were significant differences in the FODMAP content and in adherence to the MD, as well as in total energy intake and in macronutrient and micronutrient intake. In particular, the highest consumption of foods rich in FODMAPs was in Croatia in both age groups. However, the effect of FODMAPs on FGIDs did not significantly vary from one country to another. Moreover, we found no statistically significant associations between FODMAPs and FGIDs with the highest prevalence in each group.

There is evidence [19,20] that a low-FODMAP diet might have a favorable impact on IBS symptoms both in adults and children, especially abdominal pain, bloating, and diarrhea. However, it remains to be demonstrated, especially following the NICE guidelines [21], whether a low-FODMAP diet is superior to conventional IBS diets on long term follow up.

Moreover, low-FODMAP diets may have unclear consequences on gut microbiota, colonocyte metabolism and nutritional status [22,23,24]. Indeed, reducing the FODMAP content in the diet results in an important food restriction due to the elimination of many foods, such as wheat derivatives, lactose-containing dairy products, legumes and different types of fruit. Restrictive diets may be at risk of reduced intake of vitamins and micronutrients such as calcium, iron, zinc, vitamin B and vitamin D and fiber, especially in children. On the other hand, according to the results of our study, some of these micronutrients have a protective association with functional disorders (vitamin E), while fibers showed no significant association with FGIDs. Furthermore, the risk of nutritional deficiencies due to a low-FODMAP diet may be higher in persons with limited access to alternative and often expensive products present in this diet [25].

Conversely, in recent years, the protective health effects of a Mediterranean diet rich in FODMAPs have been demonstrated and populations who have adopted the diet show a remarkable reduction in all-cause mortality [26], especially from cardiovascular diseases and cancer, compared to the United States or Northern European countries [27].

In our study, good adherence to the MD was associated with a significant lower prevalence of FGIDS, in particular among both age groups, and PDS in the adolescent group, while the effect of adherence to the MD on FGIDs significantly varied from one country to another.

Several studies in adults support the beneficial effects of the MD on GI symptoms in patients with GI disease, both organic (inflammatory bowel disease) and functional (IBS, functional dyspepsia, and gastroesophageal reflux) [8,28]. However, evidence on the association between the MD and FGIDs in pediatric ages are lacking, except for a recent single-country Greek study that explored the effect of the MD on FGIDs but did not evaluate FODMAP intake [29]. In agreement with our results, the authors of that study found that a greater adherence to the MD was associated with a lower prevalence of FGIDs according to Rome III criteria. In particular, a good adherence to the MD was associated with a lower prevalence of FC. They also found that the prevalence of FGIDs was significantly higher in adolescents compared to children, and FC was s the most common disorder. Our results confirm the data from several studies exploring dietary habits in different European countries, widely demonstrating that young people exhibit the lowest level of adherence to the MD [30,31,32]. Moreover, a systematic review including 18 cross-sectional studies conducted in Mediterranean countries reported that adherence to the MD differs among countries and the pooled estimated percentage of poor adherence was 21% (confidence interval of 95% = 0.14–0.27), similar to our study [33]. Since the MD contains only a minimal amount of nutrients that can trigger GI symptoms, it has been hypothesized that the MD represents a new therapeutic strategy for patients with FGIDs. The MD might have a protective effect on FGIDs due to both the large consumption of healthy nutritional components and as a dietary model that modifies intestinal function and structure [34,35,36]. Dietary patterns represent a broader picture of food and nutrient consumption—being presented to the intestinal mucosa as a food matrix—potentially interacting with each other, and with the mucosal barrier, which may be more predictive of disease risk than individual foods or nutritional components. Furthermore, the protective effect of the MD may also be due to changes in the intestinal microbiota. Several studies have shown a reduction in *E. coli* count with a higher *Bifidobacteria* to *E. coli* ratio and higher microbial diversity, with *Prevotella* predominating over *Bacteroides* in patients with good adherence to the MD compared to subjects with low adherence [10,37,38,39,40,41].

This study has some limitations. First of all, no demographic and/or socio-economic data and anthropometric characteristics were collected. We did not analyze other factors such as family history of functional disorders, psychosocial features, and history of recent infections or stressful events that may have explained the differences between countries. Knowledge of these data could allow a better understanding of the reported intercountry differences.

Moreover, this was a parent and patient report, with related biases due to this type of study.

The major strength of our study is that we assessed the prevalence of FGIDs and the association with diet in a large cohort of pediatric subjects in different countries. Furthermore, data analysis was carried out only by the coordinating center to ensure the uniformity of the data collected.

Translated and validated R4PDQs were used to ensure a correct understanding of the various items.

## 5. Conclusions

Our findings confirm that the prevalence of FGIDs varies significantly among different Mediterranean countries, suggesting that the results of single-country studies may not be generalized. Most interestingly, the prevalence of FGIDs was not related to the FODMAP content in the diet, nor to macronutrient and micronutrient intake. However, it significantly decreased according to adherence to the MD. Further investigations on the efficacy of nutritional interventions to promote the MD and its effect on the clinical outcomes of FIGDs are warranted.

## Figures and Tables

**Table 1:** FODMAP daily intake among children (Group A) and adolescents (Group B) of all involved countries.

Country	Group A						Group B					
			FODMAP Intake (g/day)			Differences among Countries			FODMAP Intake (g/day)			Differences among Countries
	N	n. Valid	Q1	Median	Q3		N	n. Valid	Q1	Median	Q3	
Croatia	379	377	22.69	29.27	35.79	a	462	462	18.21	26.06	33.26	a
Greece	310	296	12.57	24.13	51.48	b	355	313	13.21	23.00	50.69	a
Israel	140	109	14.08	20.44	28.29	b; c	379	121	5.99	12.76	18.75	b
Italy	369	359	10.78	15.77	24.32	c	371	356	7.50	12.79	18.58	b
Macedonia	376	361	13.24	22.29	70.16	b	433	384	5.31	11.81	27.98	b
Serbia	398	384	10.08	19.67	39.24	b	450	450	5.67	10.99	40.91	b

Note: Countries sharing a letter are not significantly different.

**Table 2:** Daily intake of macronutrients and micronutrients among children (Group A) and adolescents (Group B) of all involved countries.

	Group A						Group B					
Characteristics	Croatia	Greece	Israel	Italy	Macedonia	Serbia	Croatia	Greece	Israel	Italy	Macedonia	Serbia
**Energy intake** (kcal/day), mean ± sd	1502.3 ± 377.5	1461.5 ± 466.5	1105.8 ± 266.6	1505.3± 358.7	1842.5 ± 526.9	1469.2 ± 503	1471.6 ± 480.8	1818.5 ± 493	1114.3 ± 319.6	1506.3 ± 427.5	1629.7 ± 480.5	1511.5 ± 395.1
**Macronutrients** mean ±sd												
**Total protein** (g/day)	63.3 ± 15.8	58.7 ± 18.6	51 ± 17.2	60.2 ± 14.7	72.5 ± 24.9	59.3 ± 22	62.7 ± 20.5	74.2 ± 23	54.4 ± 19	60.9 ± 17	64.5 ± 22.3	67.3 ± 74.8
**Lipids** (g/day)	53.4 ± 17.4	58.2 ± 21.1	34 ± 11	62.7 ± 20.3	74.2 ± 22.8	56.8 ± 20.4	54.6 ± 24	70.4 ± 27.4	40.7 ± 16.6	59.3 ± 20.9	60.9 ± 23.2	63.8 ± 21.1
**Carbohydrates** (g/day)	184.6 ± 49.3	185.9 ± 70.2	144.5 ± 44	185 ± 49.5	234.1 ± 83.4	190.3 ± 80.9	179.5 ± 65.5	239.4 ± 76.5	132.5 ± 48.6	193.2 ± 63.5	219 ± 80.9	183.1 ± 62.9
**Starch** (g/day)	82.9 ± 27.8	80.4 ± 47	62 ± 39.3	109 ± 9.63	127.2 ± 60.3	98.5 ± 58.9	85.3 ± 33.5	130.2 ± 57	38.2 ± 33	116 ± 46.3	135.2 ± 58.9	98.8 ± 45.3
**Cholesterol** (mg/day)	201.3 ± 90.4	199.1 ± 103.7	260.5 ± 129	194 ± 93.4	220.1 ± 112.5	213.7 ± 109.7	202.6 ± 114.1	236.1 ± 140.9	280.5 ± 192.6	198.7 ± 92.8	188.9 ± 130.1	268.9 ± 123.1
**Saturated fatty acids** (g/day)	18.6 ± 8	19.7 ± 8	12.1 ± 4.8	17.4 ± 6.6	23 ± 8.6	19.1 ± 7.4	18.7 ± 9.5	25.3 ± 12.5	12.4 ± 5.3	16.7 ± 6.3	19.9 ± 9.4	22.2 ± 7.8
**Polyunsaturated fatty acids** (g/day)	5.8 ± 3.6	6.1 ± 3.2	6.4 ± 3	6.4 ± 2.6	10.9 ± 5.8	6.5 ± 3.7	6.4 ± 5.1	7.9 ± 3.6	8.3 ± 4	6.3 ± 2.4	9.1 ± 4.8	7.7 ± 2.7
**Fiber** (g/die)	14 ± 6.2	12.2 ± 7.9	14.1 ± 6.2	12.4 ± 4.2	21.7 ± 20.9	13.5 ± 9.5	13.3 ± 6.6	18.2 ± 8.7	13 ± 8.1	12.5 ± 4.7	20.3 ± 20.3	15.2 ± 52.9
**Micronutrients** mean±sd												
**Calcium** (mg/day)	629.3 ± 224.6	608.5 ± 326.2	464.1 ± 239.2	456.9 ± 167.8	624.4 ± 265.5	523.1 ± 250.5	575.2 ± 253.7	720.9 ± 345.4	361.5 ± 181.5	438.7 ± 176.6	483.1 ± 258.7	431.9 ± 199.6
**Sodium** (mg/day)	2988 ± 562	1366.3 ± 729.7	2423.1 ± 474.8	1299.6 ± 731.6	1353.7 ± 651.8	1206.4 ± 586.6	2995.5 ± 433.7	1725 ± 946.5	7853.4 ± 451.3	1383.2 ± 743	1380.8 ± 847.4	1924.9 ± 1068.7
**Potassium** (mg/day)	2063.5 ± 857.5	1833.2 ± 652.4	4652.5 ± 129.2	1824.8 ± 491.6	2372.1 ± 1042.8	1922.1 ± 889.9	1917.9 ± 1258.7	2571.6 ± 1336.4	4325.7 ± 233.6	1800.7 ± 568	2219.9 ± 881.6	1795 ± 541.9
**Phosphorus** (mg/day)	948.7 ± 271	883.4 ± 331.6	731.5 ± 214	888.9 ± 239.1	1132.2 ± 434.6	926.7 ± 428.4	898.6 ± 316.2	1123.4 ± 385.2	723.7 ± 231.7	887.7 ± 269	999.5 ± 406.1	911.7 ± 250.8
**Iron** (mg/day)	8.2 ± 4.5	8 ± 4.3	7.6 ± 3.6	8.4 ± 2.4	12.8 ± 6.4	9.4 ± 6.1	8 ± 5	11.2 ± 4.9	7.2 ± 3.1	8.6 ± 2.8	12.2 ± 6.3	8.7 ± 3
**Zinc** (mg/day)	8 ± 2.8	7.8 ± 3.1	5.3 ± 2.1	7.9 ± 2.3	9 ± 3.5	7.6 ± 3.1	7.6 ± 3	9.1 ± 3.2	5.6 ± 2.5	7.9 ± 2.7	8.4 ± 3.4	8 ± 2.6
**Folic acid** (mcg/day)	129.8 ± 52.6	173.4 ± 122.1	198.9 ± 96.1	165.8 ± 69.9	238.7 ± 171.3	170.5 ± 128	121.4 ± 51.7	273.6 ± 186.2	195.3 ± 101	162.1 ± 74	223.8 ± 170.7	153.9 ± 78.7
**Niacin** (mg/day)	17.2 ± 7.9	10.5 ± 4.4	15.5 ± 5.4	12.5 ± 3.9	15.7 ± 8.2	12.2 ± 8.3	17 ± 7.1	14.8 ± 9	16.5 ± 10.2	12.7 ± 4.8	15 ± 7.7	12.5 ± 4.6
**Riboflavin** (mg/day)	1.2 ± 0.4	1.3 ± 0.7	1.3 ± 0.8	1.3 ± 0.9	1.5 ± 0.7	1.2 ± 0.6	1.1 ± 0.4	1.5 ± 0.6	1.4 ± 1	1.3 ± 0.8	1.3 ± 0.7	1.1 ± 0.4
**Thiamine** (mg/day)	1.7 ± 1.4	1 ± 0.5	0.8 ± 0.4	0.9 ± 0.3	1.4 ± 0.7	1 ± 0.7	1.8 ± 1.7	1.2 ± 0.5	0.7 ± 0.3	0.9 ± 0.3	1.3 ± 0.6	1.1 ± 0.7
**Vitamin A** (mcg/day)	18071 ± 300	540.8 ± 255.7	419.8 ± 242.6	603.8 ± 318.9	574 ± 369.2	484.6 ± 235.8	13706.6 ± 285.7	678.7 ± 420.2	305.7 ± 177	534.6 ± 327.1	449.5 ± 274	766.4 ± 215.2
**Vitamin B6** (mg/day)	1.3 ± 0.7	1.4 ± 0.8	1 ± 0.4	1.4 ± 0.5	1.8 ± 0.9	1.3 ± 0.8	1.2 ± 0.5	1.7 ± 0.7	1.2 ± 0.7	1.4 ± 0.5	1.5 ± 0.8	1.2 ± 0.4
**Vitamin C** (mg/day)	248.3 ± 230	97 ± 65.7	41.8 ± 38	75.2 ± 49.6	98.5 ± 72.4	91.6 ± 63.4	69.8 ± 45.4	94.3 ± 79.3	38.1 ± 35.7	74.4 ± 52.4	79.2 ± 66.2	65.1 ± 45.7
**Vitamin D** (mcg/day)	1.2 ± 1.8	3.4 ± 2	2.5 ± 2.3	2.3 ± 1.7	2.6 ± 1.7	2.7 ± 1.6	1.4 ± 2.5	3.3 ± 2.2	2.5 ± 2.4	2.4 ± 1.9	2.1 ± 1.7	2.5 ± 1.6
**Vitamin E** (mg/day)	5.4 ± 2.6	6.4 ± 3	5.8 ± 5.8	8.7 ± 3.8	8.7 ± 3.5	5.7 ± 3.4	5.5 ± 3.1	7.4 ± 3.2	5.5 ± 2.9	7.6 ± 4	7.2 ± 3.7	5.5 ± 2.5

## Data Availability

Not applicable.

## Supplementary Materials

The following supporting information can be downloaded at: https://www.mdpi.com/article/10.3390/nu14112335/s1, Table S1: Overall FGIDs Prevalence in Children (Group A) and Adolescents (Group B); Table S2: Valid case and prevalence (%) of each functional gastrointestinal disorder in children (Group A) and adolescents (Group B), in all involved countries.

## Abbreviations

FGIDs: functional gastrointestinal disorders; IBS: irritable bowel syndrome; FODMAPs: fermentable oligosaccharides, disaccharides, monosaccharides, and polyol; MD: Mediterranean diet; R4PDQ: Rome IV Diagnostic Questionnaire on Pediatric Functional Gastrointestinal Disorders; KIDMED: Mediterranean Diet Quality Index in Children and Adolescents; FC: functional constipation; PDS: postprandial distress syndrome; AM: abdominal migraine; EPS: epigastric pain syndrome.
