# Supplementary material for: Diet and Pediatric Functional Gastrointestinal Disorders in Mediterranean Countries
Source: Nutrients. 2022 Jun 2;14(11):2335. doi: 10.3390/nu14112335 (PMC9183175; doi:10.3390/nu14112335)
Supplement: Supplementary file 1 [file nutrients-14-02335-s001.zip › nutrients-1717852-supplementary.pdf]

Supplementary Table S1: Overall FGIDs Prevalence in Children (Group A) and Adolescents (Group B)

	Group A<br>n=1972<br>mean age:7.3 ± 1.6y<br>F 53%			Group B<br>n=2450<br>mean age:13.1 ± 2.3 y<br>F 57.6%		
	**Valid case**	**n (%)**	**95%CI**	**Valid case**	**n (%)**	**95%CI**
**Cyclic vomiting Syndrome**	1901	3 (0.16%)	(0.03; 0.46)	2370	10 (0.42%)	(0.20; 0.77)
**Functional Nausea**	1823	0		2232	10 (0.45%)	(0.22; 0.82)
**Functional Vomiting**	1886	2 (0.11%)	(0.01; 0.38)	2360	9 (0.38%)	(0.17; 0.72)
**Adolescent Rumination Syndrome**	1840	1 (0.05%)	(0.01; 0.30)	2340	10 (0.43%)	(0.21; 0.78)
**Aerophagia**	1843	7 (0.38%)	(0.15; 0.78)	2355	9 (0.38%)	(0.17; 0.72)
**Postprandial Distress Syndrome**	1642	55 (3.35%)	(2.53; 4.34)	2198	118 (5.37%)	(4.46; 6.39)
**Epigastric Pain Syndrome**	1619	4 (0.25%)	(0.07; 0.63)	2144	25 (1.17%)	(0.76; 1.72)
**Irritable Bowel Syndrome**	1793	11 (0.61%)	(0.31; 1.09)	2250	25 (1.11%)	(0.72; 1.64)
**Abdominal Migraine**	1877	21 (1.12%)	(0.69; 1.71)	2347	65 (2.77%)	(2.14; 3.52)
**Functional Abdominal Pain-NOS**	1850	11 (0.59%)	(0.30; 1.06)	2333	9 (0.39%)	(0.18; 0.73)
**Functional Constipation**	1930	144 (7.46%)	(6.33; 8.73)	2423	331 (13.7%)	(12.3; 15.1)
**Nonretentive Fecal Incontinence**	1883	0	-	2294	0	-

Note: Functional Abdominal Pain-NOS: Functional Abdominal Pain-Not Otherwise Specified

Supplementary Table S2. Valid case and prevalence (%) of each functional gastrointestinal disorder in children (Group A) and adolescents (Group B), in all involved countries

	GROUP A						GROUP B					
**FGIDs, (valid case), % prevalence**	**Croatia n=379**	**Greece n=310**	**Israel n=140**	**Italy n=369**	**Macedonia n=376**	**Serbia n=398**	**Croatia n=462**	**Greece n=355**	**Israel n=379**	**Italy n=371**	**Macedonia n=433**	**Serbia n=450**
**FGIDs**	(292) 12%	(270) 18%	(75) 33%	(239) 26%	(226) 12%	(332) 8%	(308) 18%	(348) 16%	(319) 50%	(263) 26%	(280) 33%	(365)18%
**Cyclic vomiting Syndrome**	(370)0	(309)0.32%	(130)0	(132)0	(365)0.55%	(395)0	(449)0.45%	(350)0	(374)1.34%	(345)0	(412)0.24%	(440)0.45%
**Functional Nausea**	(363)0	(306)0	(128)0	(414)0	(321)0	(391)0	(430)0.47%	(341)0	(351)0.57%	(330)0.91%	(345)0.29%	(435)0.46%
**Functional Vomiting**	(367)0	(309)0	(129)0	(332)0.30%	(360)0.28%	(389)0	(459)0	(349)0	(373)1.88%	(345)0.58%	(408)0	(435)0
**Adolescent Rumination Syndrome**	(362)0	(307)0	(107)0	(325)0.31%	(345)0	(394)0	(438)0.68%	(348)0	(368)1.90%	(346)0	(400)0	(440)0
**Aerophagia**	(371)0.54%	(303)0	(126)0	(313)0.96%	(344)0.58%	(386)0	(447)0	(350)0	(368)0.54%	(349)0.57%	(396)0.51%	(445)0.67%
**Postprandial Distress Syndrome**	(320)4.69%	(275)3.27%	(96)12.50%	(271)4.43%	(292)0.34%	(388)1.55%	(394)5.84%	(320)2.81%	(356)12.08%	(322)6.52%	(375)2.67%	(431)2.78%
**Epigastric Pain Syndrome**	(314)0.64%	(274)0	(93)0	(268)0.75%	(283)0	(387)0	(388)0.77%	(319)0.31%	(343)1.46%	(314)2.55%	(356)1.40%	(424)0.71%
**Irritable Bowel Syndrome**	(356)1.69%	(296)0	(130)5.38%	(301)0.33%	(331)1.81%	(379)0.26%	(431)0.93%	(339)0.88%	(346)3.47%	(323)0.62%	(379)0.26%	(432)0.69%
**Abdominal Migraine**	(364)0.55%	(304)0.66%	(134)1.49%	(329)0.61%	(360)0.83%	(386)2.59%	(442)1.13%	(345)1.74%	(366)9.84%	(342)1.46%	(419)2.86%	(433)0.23%
**Functional Abdominal Pain-NOS**	(361)1.11%	(302)0.33%	(132)0.76%	(318)0.63%	(344)0.29%	(393)0.51%	(436)0.23%	(344)0.29%	(364)0.82%	(339)0.59%	(407)0	(443)0.45%
**Functional Constipation**	(378)4.50%	(310)13.55%	(136)5.88%	(338)13.90%	(370)5.95%	(398)2.01%	(358)6.55%	(354)11.02%	(373)26.81%	(368)10.33%	(420)17.38%	(450)11.33%
**Nonretentive Fecal Incontinence**	(376)0	(305)0	(136)0	(317)0	(354)0	(395)0	(449)0	(348)0	(363)0	(301)0	(394)0	(439)0

Note: Functional Abdominal Pain-NOS: Functional Abdominal Pain-Not Otherwise Specified
